# Supplementary figures and images for: Bacterial contact induces polar plug disintegration to mediate whipworm egg hatching
Source: PLoS Pathog. 2023 Sep 22;19(9):e1011647. doi: 10.1371/journal.ppat.1011647 (PMC10550136; doi:10.1371/journal.ppat.1011647)

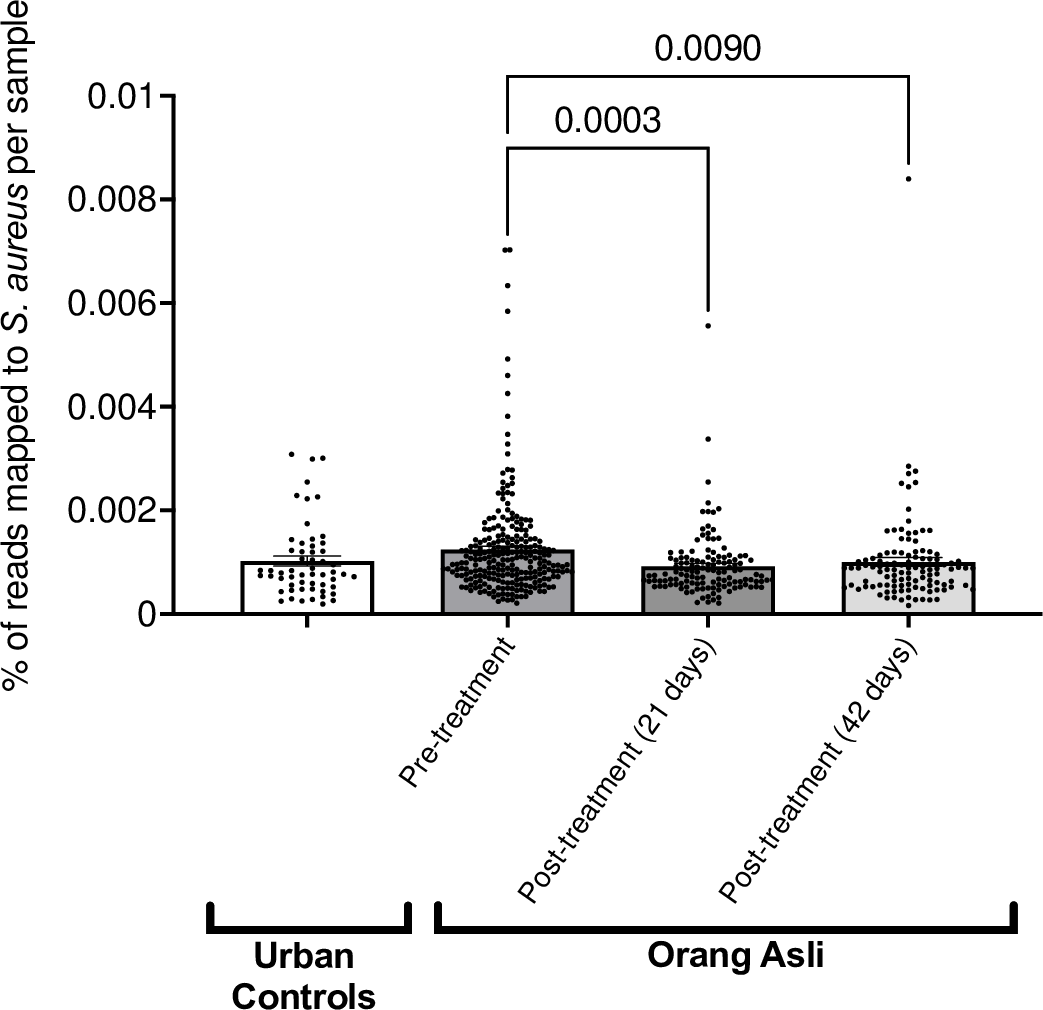

Supplement: S1 Fig — Percent reads mapped to S. aureus from stool samples collected from people in the urban control group, Orang asli prior to treatment with anthelmintics, and Orang Asli 21 and 42 days post-anthelmintic treatment. Dots represent percent prevalence of S. aureus in stool from a single individual. Bars show means and SEM. Kruskal-Wallis test followed by a Dunn’s multiple comparisons test was used. (TIF) [file ppat.1011647.s001.tif]

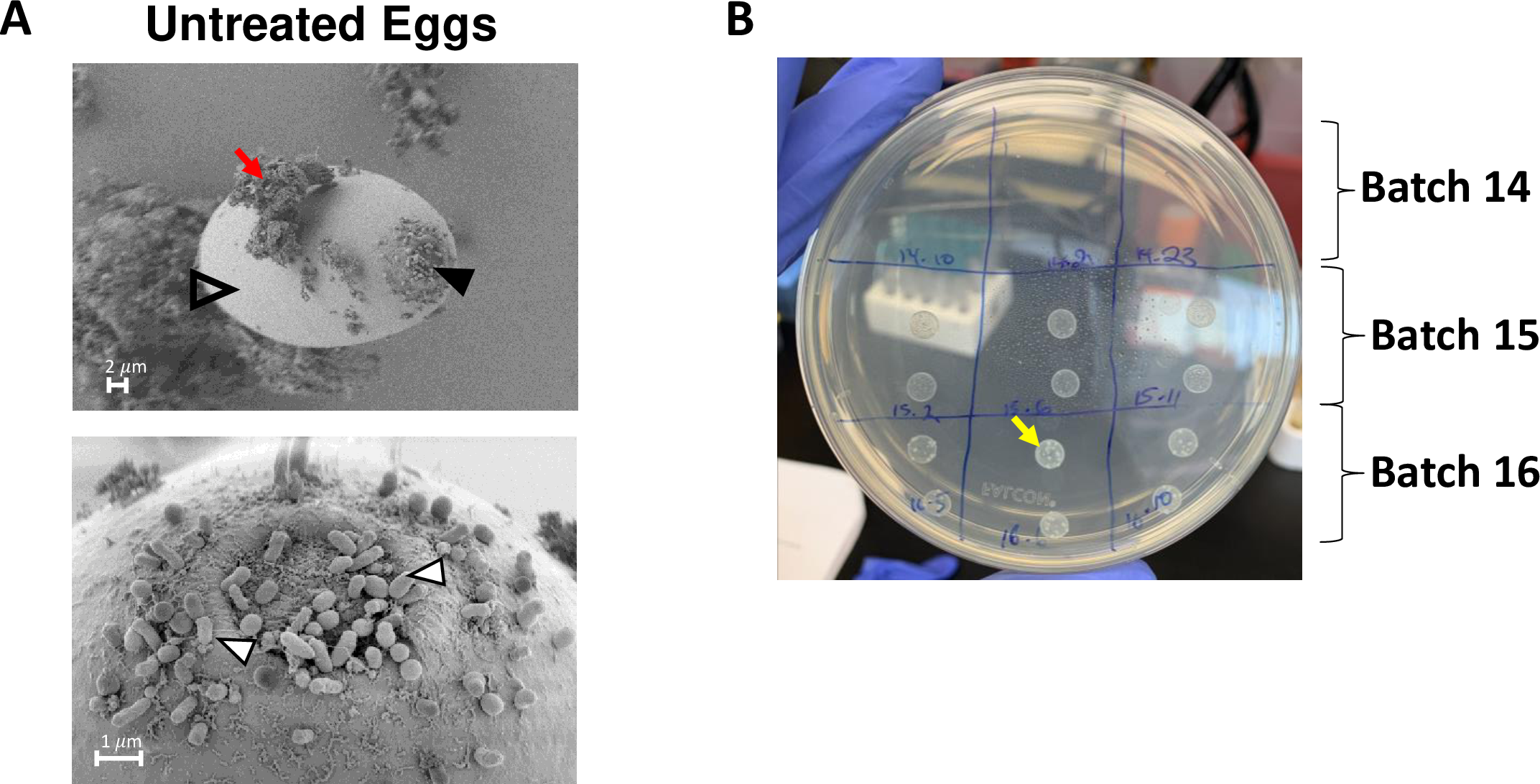

Supplement: S2 Fig — (A) Representative low (top) and high magnification (bottom) SEM images of T. muris eggs (clear arrowhead) that were untreated with bacteria. White arrowheads correspond to bacteria on polar plug regions of the eggs denoted by black arrowheads. Red arrow corresponds to debris present on egg suface. (B) Image of LB plate with overnight bacterial growth (yellow arrow) from 3 different batches of eggs (batch 14, 15, and 16). (TIF) [file ppat.1011647.s002.tif]

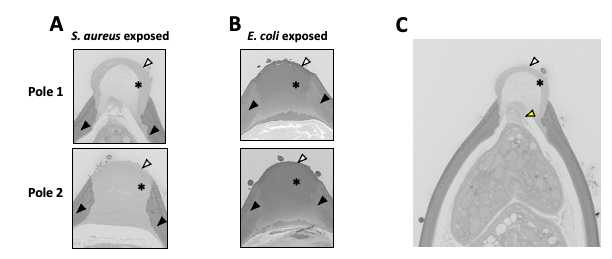

Supplement: S3 Fig — (A, B) Representative electron micrograph of a section of polar plugs (black asterisk) on eggs exposed to S. aureus (left) or E. coli (right). Images of Pole 1 (top) and Pole 2 (bottom) were collected from the same egg. Outer vitelline layer is denoted by white arrowheads and eggshell is denoted by black arrowheads. (C) Representative electron micrograph of a section of polar plug (black asterisk) on an egg exposed to S. aureus. Outer vitelline layer is denoted by the white arrowhead and point of contact between inner surface of the plug and the larva is denoted by the yellow arrowhead. (TIFF) [file ppat.1011647.s003.tiff]

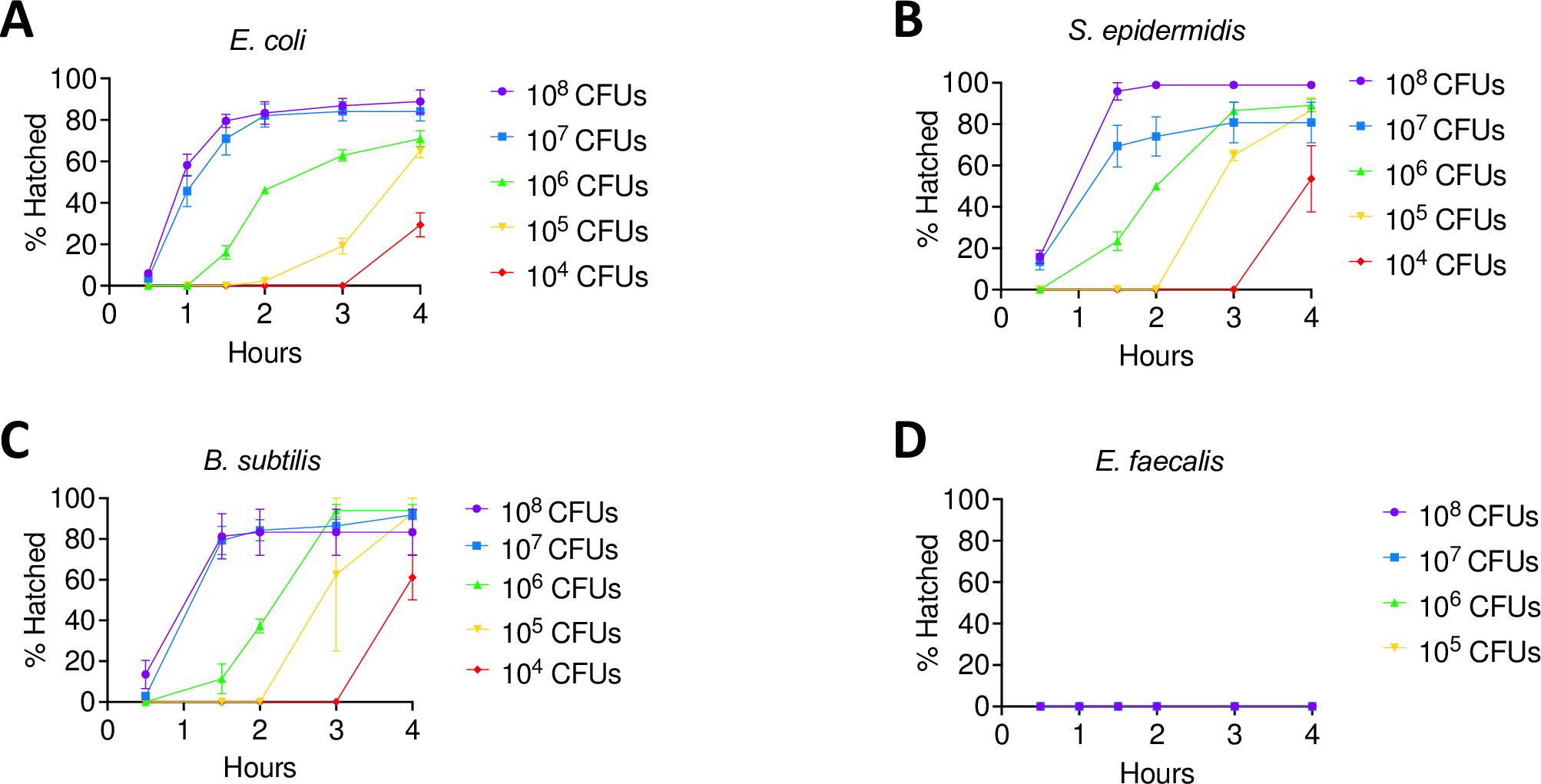

Supplement: S4 Fig — (A-D) Percent of T. muris eggs hatched after incubation with 10-fold dilutions of overnight E. coli (A), S. epidermidis (B), B. subtilis (C) and E. faecalis (D) culture ranging from approximately 104–108 CFU. Data points and error bars represent mean and SEM. (TIF) [file ppat.1011647.s004.tif]
